# Fibroblast Growth Factor 21 Improves Insulin Sensitivity and Synergizes with Insulin in Human Adipose Stem Cell-Derived (hASC) Adipocytes

**DOI:** 10.1371/journal.pone.0111767

**Published:** 2014-11-03

**Authors:** Darwin V. Lee, Dongmei Li, Qingyun Yan, Yimin Zhu, Bryan Goodwin, Roberto Calle, Martin B. Brenner, Saswata Talukdar

**Affiliations:** Cardiovascular Metabolic and Endocrine Diseases (CVMED) Research Unit, Pfizer Worldwide Research & Development, Cambridge, MA, United States of America; University of Minnesota - Twin Cities, United States of America

## Abstract

Fibroblast growth factor 21 (FGF21) has evolved as a major metabolic regulator, the pharmacological administration of which causes weight loss, insulin sensitivity and glucose control in rodents and humans. To understand the molecular mechanisms by which FGF21 exerts its metabolic effects, we developed a human in vitro model of adipocytes to examine crosstalk between FGF21 and insulin signaling. Human adipose stem cell-derived (hASC) adipocytes were acutely treated with FGF21 alone, insulin alone, or in combination. Insulin signaling under these conditions was assessed by measuring tyrosine phosphorylation of insulin receptor (InsR), insulin receptor substrate-1 (IRS-1), and serine 473 phosphorylation of Akt, followed by a functional assay using 14C-2-deoxyglucose [^14^C]-2DG to measure glucose uptake in these cells. FGF21 alone caused a modest increase of glucose uptake, but treatment with FGF21 in combination with insulin had a synergistic effect on glucose uptake in these cells. The presence of FGF21 also effectively lowered the insulin concentration required to achieve the same level of glucose uptake compared to the absence of FGF21 by 10-fold. This acute effect of FGF21 on insulin signaling was not due to IR, IGF-1R, or IRS-1 activation. Moreover, we observed a substantial increase in basal S473-Akt phosphorylation by FGF21 alone, in contrast to the minimal shift in basal glucose uptake. Taken together, our data demonstrate that acute co-treatment of hASC-adipocytes with FGF21 and insulin can result in a synergistic improvement in glucose uptake. These effects were shown to occur at or downstream of Akt, or separate from the canonical insulin signaling pathway.

## Introduction

Fibroblast growth factor 21 (FGF21) is an atypical member belonging to the fibroblast growth factor family, and serves as an endocrine hormone, which was originally identified to increase glucose uptake in fat cells [1]. In mice, FGF21 is primarily produced in the pancreas, liver and brown adipose tissue [2]. The physiological role of FGF21 is to modulate metabolic processes required for adapting the body to starvation, whereas pharmacological administration of FGF21 serves as a potent antidiabetic agent in mice [3]. Multiple reports have demonstrated that pharmacological administration of FGF21 in obese, diabetic mice improves insulin sensitivity and glycemic control, ameliorates dyslipidemia, promotes energy expenditure, and causes weight loss [4]–[7].

The metabolic benefits of pharmacologically administered FGF21 are conferred through its action on the receptor, FGF receptor 1c (FGFR1c), and its obligate co-receptor, b-klotho (KLB) [8], [9]. Adipose tissue is regarded as one of the primary target tissues of FGF21 action, since it expresses both FGFR1c and KLB [2], [8], [9], and lipodystrophic mice are refractory to the metabolic benefits of pharmacological FGF21 administration [10]. Moreover, adipocyte-specific deletion of FGFR1 or KLB ameliorates the metabolic effects of pharmacological FGF21 administration [3], [9]. Given that both FGFR1 and KLB are required for FGF21 action [9], and since there is negligible expression of FGFR1c in mouse liver [2], one can hypothesize that FGF21 does not have a direct effect on the liver [11]. Indeed, studies have shown that adiponectin is required for glycemic control and insulin sensitizing effects of pharmacological FGF21 administration in obese mice [12], [13]. Importantly, both FGFR1c and KLB are required for FGF21 action since the loss of either of these proteins abolishes the metabolic effects of FGF21 [8], [9].

In obese mouse models, acute FGF21 administration was shown to cause rapid decrease in blood glucose levels and improved glucose tolerance and insulin sensitivity. These effects are associated with an increase in FGF21 signaling in liver and adipose tissue, indicating that acute glucose-lowering and insulin-sensitizing effects of FGF21 are potentially associated with its metabolic actions in liver and adipose tissues [14]. In primary human adipocytes, FGF21 decreases hormone-stimulated lipolysis by decreasing expression of the lipid droplet-associated phosphoprotein perilipin, without affecting differentiation of these cells and expression of genes involved in modulating lipolysis [15].

Due to the beneficial effects of FGF21 in experimental models, FGF21 is regarded as a potentially promising therapeutic agent for the treatment of metabolic disorders such as obesity and diabetes [16]. Administration of FGF21 to humans, recapitulates many of the effects observed in rodents, including reduction in body weight and circulating lipids, and modest improvements in glycemic control improvements in plasma lipid profile, and modest improvement in glycemic control [17]. Since adipose tissue is the primary target tissue for FGF21 action, we sought to explore the mechanisms by which FGF21 modulates adipocyte function by using human adipose stem cell derived cells (hASC) that were differentiated into mature adipocytes. This in vitro cell culture system has been shown to exhibit many of the characteristics of mature human adipocytes [18]. We demonstrate that these hASCs can differentiate into adipocytes and exhibit similar levels of expression of FGFR1 and KLB as native human adipocytes. In this hASC adipocyte model, we show that FGF21 synergizes with insulin to increase downstream markers of insulin signaling, suggesting a role for acute insulin sensitizing effects of FGF21 in man. To our knowledge, this is the first report showing not only a synergy between FGF21 and insulin signaling but also an interplay between these two hormones in regulating glucose disposal in adipose cells through a mechanism that is distinct from the canonical insulin signaling pathway.

## Results

### Expression of FGFR1 and KLB in hASC-adipocytes is similar to human adipose tissue

**Figure 1:**
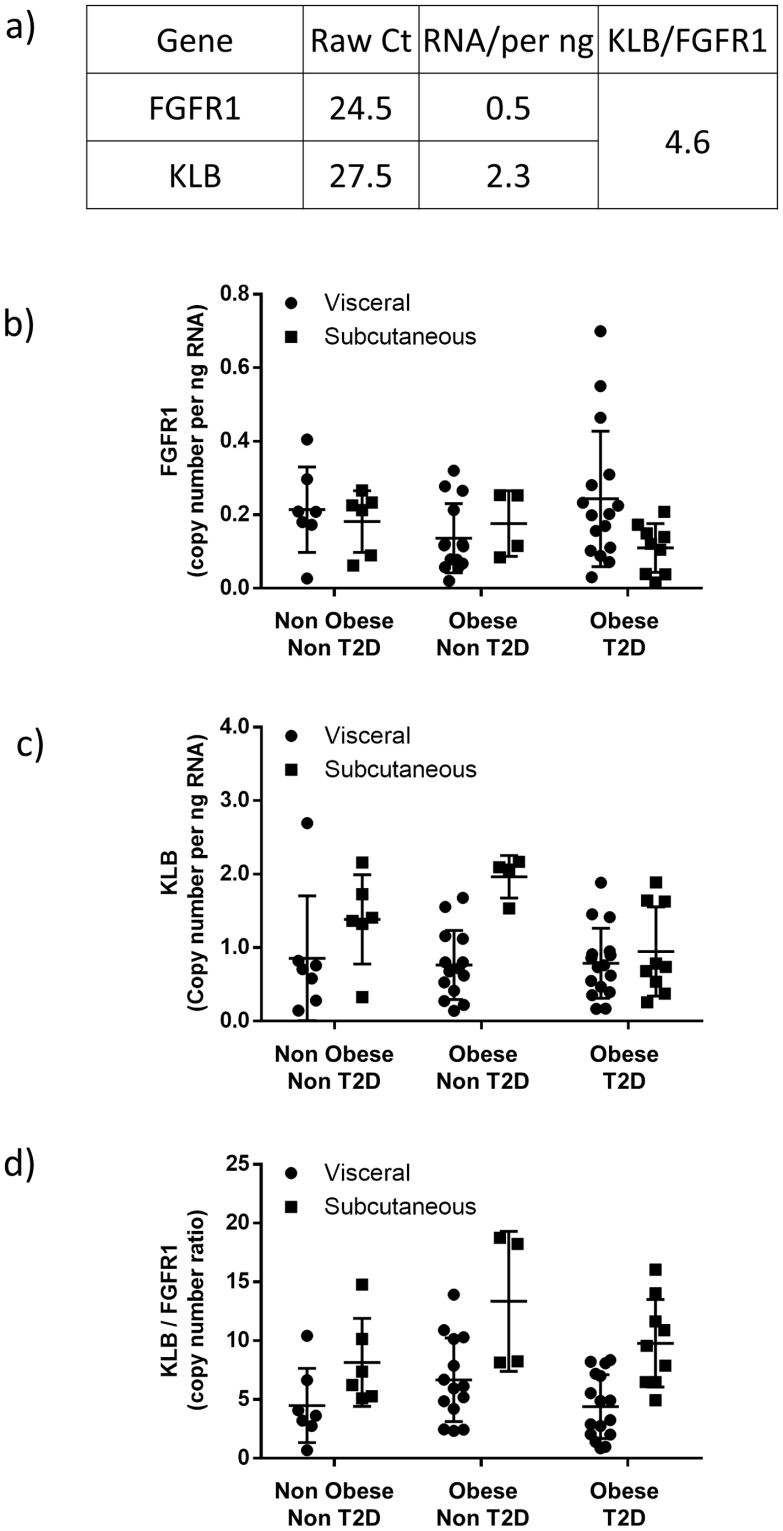
Expression of FGFR1 and KLB are consistent in hASCs and human adipose tissue. FGFR1 and KLB mRNA in a) hASC-adipocytes, b) human subcutaneous (SC) and visceral (Vis) adipose tissue. c) Ratio of KLB:FGFR1 in human SC and VIS adipose tissue. n = 7 lean normal, n = 6 obese non T2D and n = 10 obese T2D.

Previous reports have shown FGFR1 and KLB expression in 3T3L1 cells and mouse adipose tissue [2]. We assessed the expression of FGR1c and KLB in hASC differentiated into adipocytes. hASC-adipocytes express both FGFR1 and KLB (Fig. 1a). To confirm whether the expression is similar in man, we obtained subcutaneous (SC) and visceral (Vis) adipose tissue samples from non obese non type 2 diabetic (T2D), obese non T2D, and obese T2D subjects. FGFR1 (Fig. 1b) and KLB (Fig. 1c) are expressed in both Vis and SC adipose tissue. Copy number of FGFR1 is approximately 0.2 in both adipose depots across subject cohorts (Fig. 1b). Copy number of KLB is approximately 0.9 in visceral depot of all subject cohorts and SC depot of obese T2D subjects (Fig. 1c), whereas copy number in SC depot of non obese non T2D subjects and obese non T2D subjects is approximately 1.5 and 2 respectively (Fig. 1c). Expression of KLB and FGFR1 in both adipose depots is similar across subject cohorts. To precisely quantitate the expression of these genes, we determined copy number of FGFR1 and KLB in both hASC-adipocytes and human adipose tissue. The ratio of KLB:FGFR1 is 4.6∶1 in hASCs (Fig. 1a) and between 4.8 to 5.2 in both SC and Vis depot of lean healthy subjects and Vis depot of obese T2D subjects (Fig. 1c). The KLB:FGFR1 ratio is approximately 6 in both adipose depots of obese non diabetic subjects and SC depot of obese T2D subjects (Fig. 1c). Overall, the expression ratio of KLB and FGFR1 is similar in hASC-adipocytes and human adipose tissue suggesting that these cells could serve as a useful model to dissect FGF21 signaling.

### FGF21 increases glucose uptake in hASC-adipocytes

**Figure 2:**
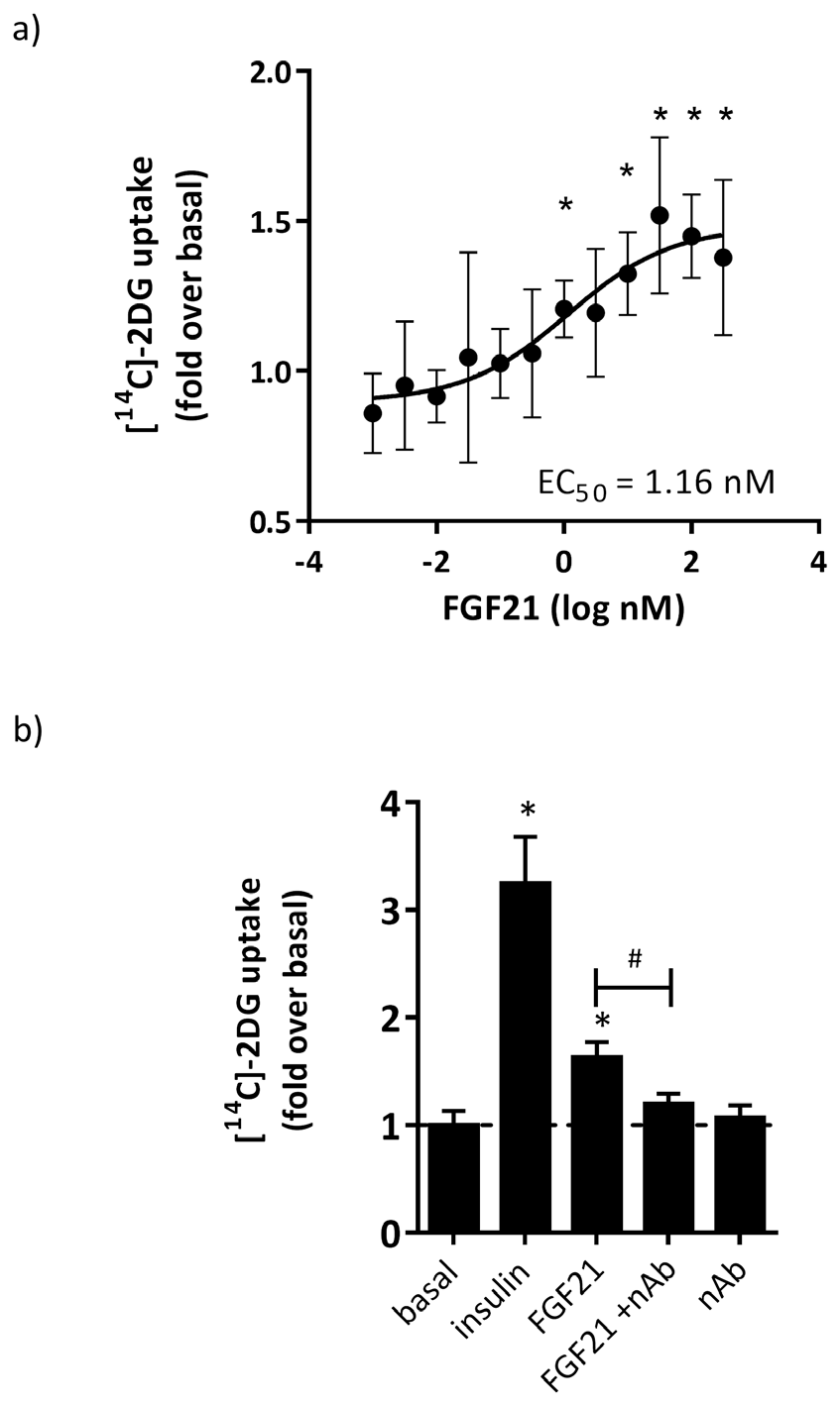
FGF21 modestly induced glucose uptake, which was inhibited by anti-FGF21 neutralizing antibody. hASC-adipocytes were treated with FGF21 for 30 minutes prior to the addition of [14C]-2DG for 30 min. a) Dose dependent effects of FGF21 on glucose uptake. N = 8 wells+S.D.; EC50 of 1.16 nM and max induction of 65% above basal signal. b) Effects of a FGF21 neutralizing antibody on the FGF21-dependent effects on glucose uptake. N = 6 wells+S.D.; induction with 32 nM FGF21 at 63% above basal, which is significantly inhibited by co-treatment with 100 ug/ml anti-FGF21 neutralizing antibody (nAb) (# p<0.05). * p<0.05 vs basal.

To determine the effects of FGF21 on glucose uptake hASC-adipocytes, we treated cells with various concentrations of native human FGF21 (0.001 to 320 nM) for 30 min, followed by the addition of ^14^C-labeled 2-deoxyglucose (2DG) in order to assess glucose uptake. FGF21 treatment dose-dependently increased ^14^C-2-deoxyglucose [^14^C]-2DG uptake in hASC-adipocytes with an EC_50_ of 1.16 nM with a maximum induction of approximately 65% above basal (Fig. 2a). To confirm if the observed glucose uptake was mediated by FGF21, we pre-incubated FGF21 (32 nM) with 100 µg/ml of anti-FGF21 neutralizing antibody (nAb) and observed that nAb significantly inhibited the effects of FGF21 on glucose uptake (Fig. 2b) demonstrating the specificity of effect. nAb alone did not have any effect on glucose uptake in these cells.

### FGF21 increases phosphorylation of proteins in the insulin signaling pathway

**Figure 3:**
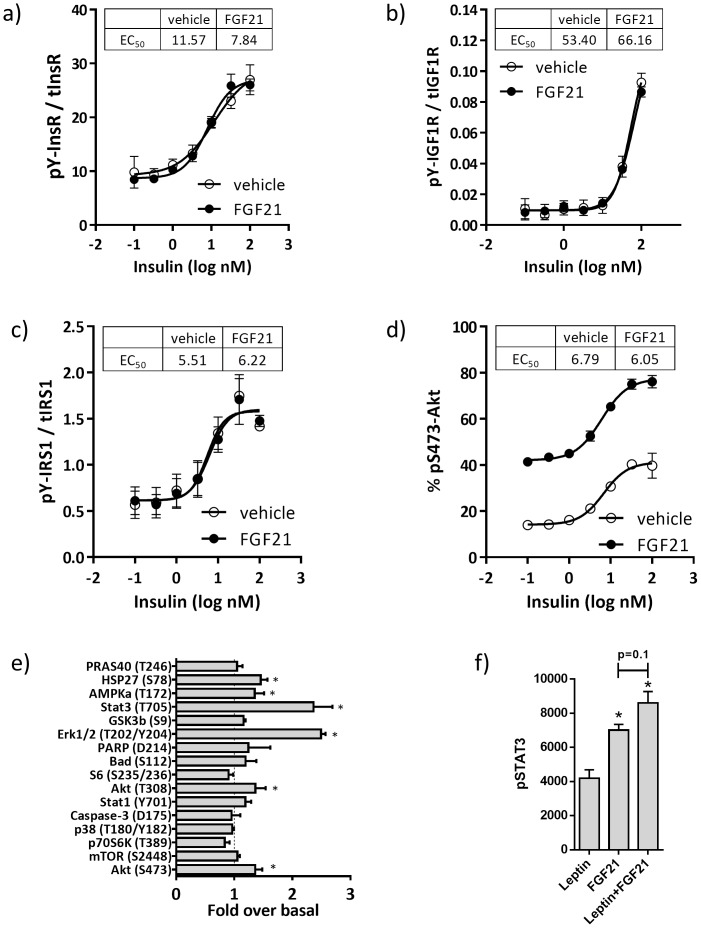
Activation of the insulin signaling pathway by FGF21 occurs downstream of IRS-1. hASC-adipocytes were treated with insulin, without (veh, open-circles) or with 100 nM FGF21 (closed-circles) for 5 min, then assayed for a) insulin receptor tyrosine phosphorylation, b) IGF1R tyrosine phosphorylation, c) IRS1 tyrosine phosphorylation, and d) Akt serine-473 phosphorylation. The level of each phosphoprotein was normalized to their respective total protein content. Each value represents mean±S.D. from 3 replicate wells. d) Effects of insulin with or without FGF21 cotreatment on signaling. Insulin EC_50_ is displayed. e) PathScan analyses of intracellular proteins in absence and presence of 100 nM FGF21 treated-hASC-adipocytes for 10 min. *p<0.05 vs basal. f) Phosphorylation of STAT3 upon treatment with leptin alone, FGF21 alone, or in combination.

To determine if FGF21 modulated insulin signaling in these cells, we treated hASC-adipocytes with various concentrations of insulin (0.001 to 320 nM) in absence and presence of 100 nM FGF21 for 5 min and assayed for tyrosine phosphorylation of the insulin receptor (pY-InsR, Fig. 3a), IGF1R (pY-IGFR1, Fig. 3b), IRS1 (pY-IRS1, Fig. 3c) and Akt S473 phosphorylation (pS473-Akt, Fig. 3d). FGF21 co-treatment had no effect on insulin EC_50_ values for any of these analytes. However, FGF21 treatment increased basal and maximum pS473-Akt, an effect which was additive to the effects of insulin alone (Fig. 3d).

To interrogate whether other proteins are associated with FGF21 action in the adipocytes, we performed intracellular signaling using PathScan from Cell Signaling, that allows for simultaneous detection of 18 important and well-characterized signaling molecules when phosphorylated or cleaved. Consistent with previous reports [10], FGF21 increased pERK in hASC-adipocytes. Surprisingly, FGF21 also increased STAT3 phosphorylation in these cells compared to control (Fig. 3e). Interestingly, FGF21 treatment also caused a modest, but significant increase in phosphorylation of AMPKα and HSP27 (Fig. 3e). Since phosphorylated STAT3 is a marker for leptin action, we wanted to determine the effects of FGF21 on P-STAT3 in these cells in the absence and presence of leptin. FGF21 increased P-STAT3 to a greater extent than leptin alone, and trended higher in combination with leptin (Fig. 3f), suggesting an additive effect, similar to that observed with Akt phosphorylation.

### Interaction of intact C-terminal of FGF21 and KLB is required for FGF21 signaling in hASC-adipocytes

**Figure 4:**
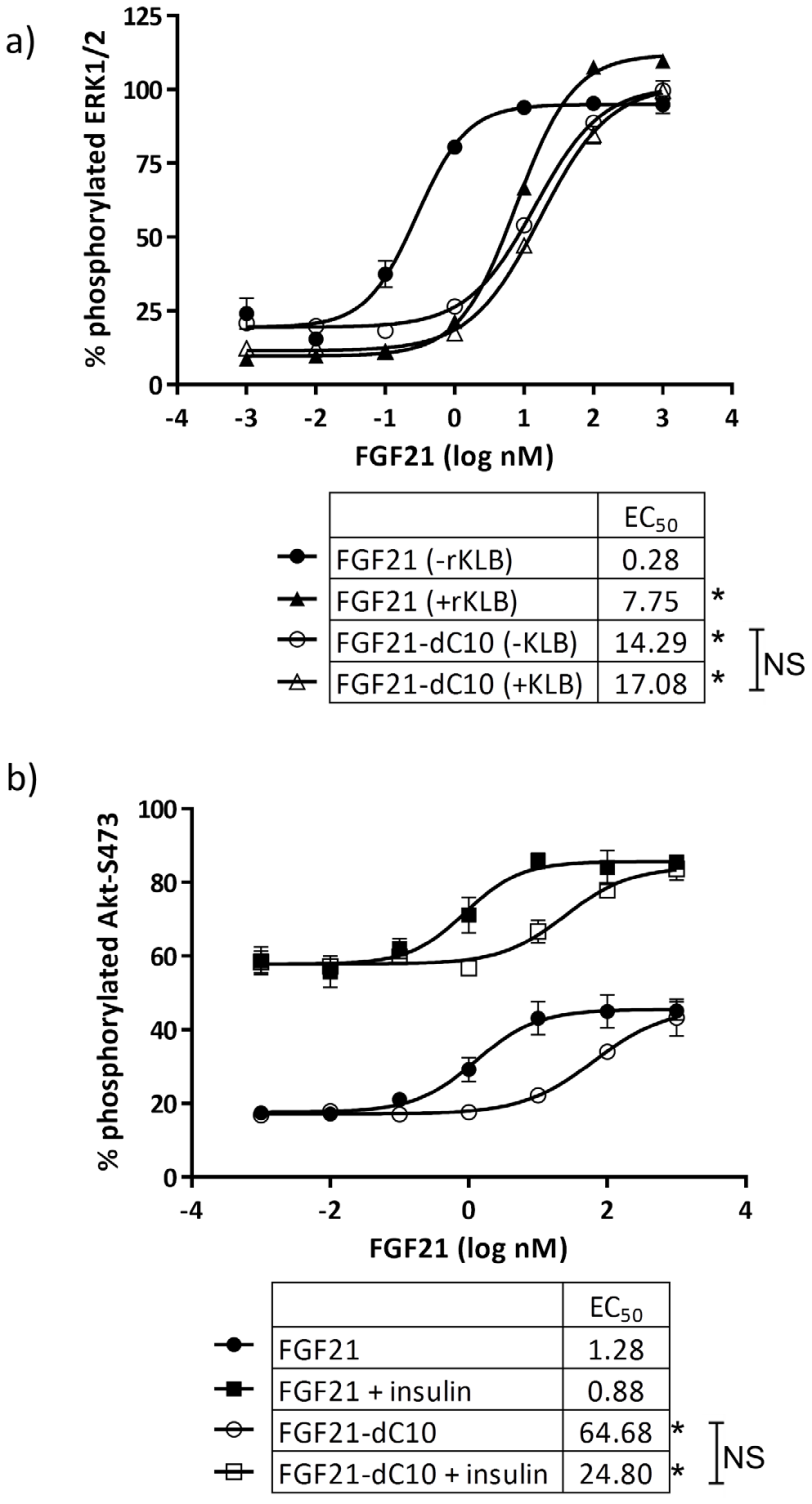
The C-terminal beta-klotho binding domain of FGF21 influenced the phosphorylation of S473-Akt by insulin and FGF21 co-treatment. hASC-adipocytes were treated with various concentrations of FGF21 (closed-shapes) or FGF21 dC10 (open-shapes), without (circles) or with 100nM insulin (squares) for 5 min, then assayed for S473-Akt phosphorylation normalized to total Akt. Data are mean±SD for 3 replicate. * p<0.05 FGF21 vs FGF21 dC10. * Significant at p<0.05.

Previous reports [19], [20] have shown that deletion of the C-terminal domain of FGF21 abrogated its binding efficiency to KLB and diminished its potency for increasing ERK phosphorylation in 293T cells overexpressing KLB, and in 3T3L1 adipocytes. To confirm whether FGF21 signaling in hASC-adipocytes was dependent on interaction of intact FGF21 and KLB, we compared the potency of native FGF21 to a recombinant FGF21 protein with a 10 amino acid deletion in the C-terminus (FGF21-dC10) for their ability to increase phosphorylation of ERK and Akt. Treatment of hASC-adipocytes with various concentrations of intact FGF21 or FGF21-dC10 for 10 min demonstrated that deletion of the last 10 amino acids of FGF21 shifted the EC_50_ for pERK by about 50-fold (Fig. 4a). Pre-treatment of FGF21 with 100 nM recombinant human KLB (rhKLB) also significantly reduced the potency of FGF21 for pERK by about 27-fold but had no effect on the potency of FGF21-dC10 which lacks the KLB binding domain (Fig. 4a). These results demonstrate that consistent with observations in rodent and overexpressing cells, interaction of intact C-terminal of FGF21 with KLB is required for signaling in hASC-adipocytes. The 10 amino acid C-terminal deletion similarly shifted the potency of FGF21 for Akt phosphorylation by about 50-fold, but interestingly, this effect was not influenced by co-treatment with 100 nM insulin, suggesting the additive effects of insulin and FGF21 on Akt phosphorylation are likely driven via independent signaling pathways that converge at Akt (Fig. 4b).

### Intact FGF21 improves adipocyte insulin sensitivity

**Figure 5:**
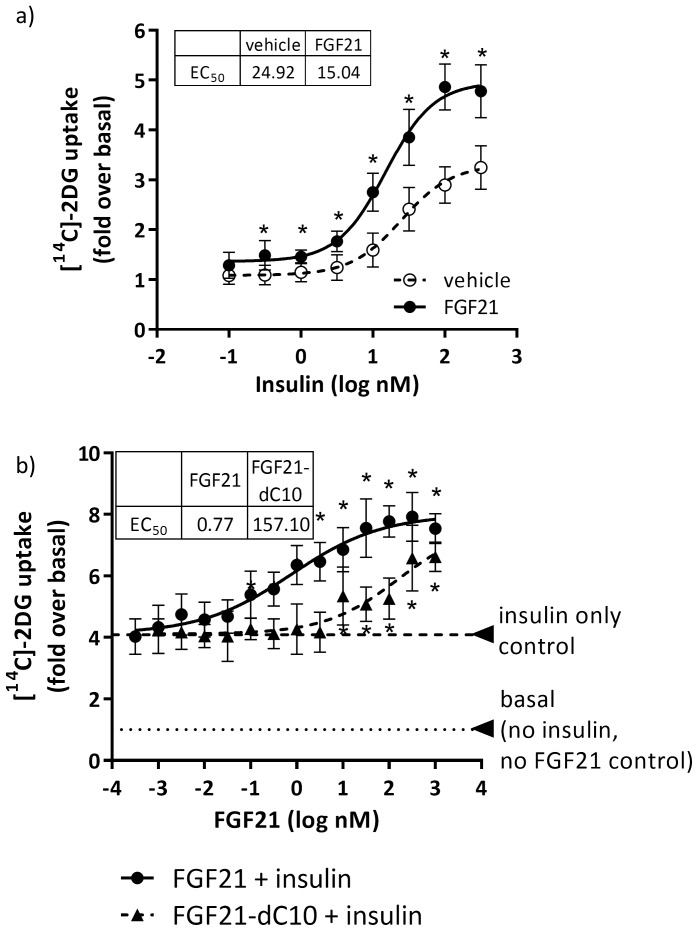
Synergistic induction of glucose uptake by FGF21 and insulin co-treatment was influenced by the C-terminal beta-klotho binding domain of FGF21. **a**) hASC-adipocytes were treated with insulin, with or without 100 nM FGF21, for 30 minutes, then spiked with [^14^C]-2DG for 30 min. * p<0.05 vs vehicle. **b**) hASC-adipocytes were treated with FGF21 or FGF21 dC10, with or without 100 nM insulin, for 30 minutes, then spiked with [14C]-2DG for 30 min. * p<0.05 vs 100 nM insulin only. All data are mean±SD (n = 8).

To determine if the additive effect of insulin and FGF21 on Akt phosphorylation translated downstream to additive effects on glucose uptake, we treated hASC-adipocytes with various concentrations of insulin in the absence and presence of 100 nM FGF21 for 30 minutes prior to the addition of [^14^C]-2DG for the assessment of glucose uptake. FGF21 alone caused minimal basal glucose uptake compared to vehicle but in presence of insulin, FGF21 increased maximal glucose uptake by 2-fold compared to insulin alone (Fig. 5a). To confirm that this synergistic effect is specific to FGF21 signaling, we treated hASC-adipocytes with various concentrations of FGF21 or FGF21-dC10 and insulin (100 nM) for 30 min prior to the addition of [^14^C]-2DG and assessed glucose uptake. We observed that both FGF21 and FGF21-dC10 increased insulin-dependent glucose uptake in a dose-dependent manner. However, FGF21-dC10 was about 200-fold less potent compared to native FGF21 (Fig. 5b), suggesting the synergistic effects of intact FGF21 on insulin-mediated glucose uptake is dependent on interaction with KLB.

### FGF21 and insulin synergistically increased phosphorylation of Akt and ATM/ATR substrates

**Figure 6:**
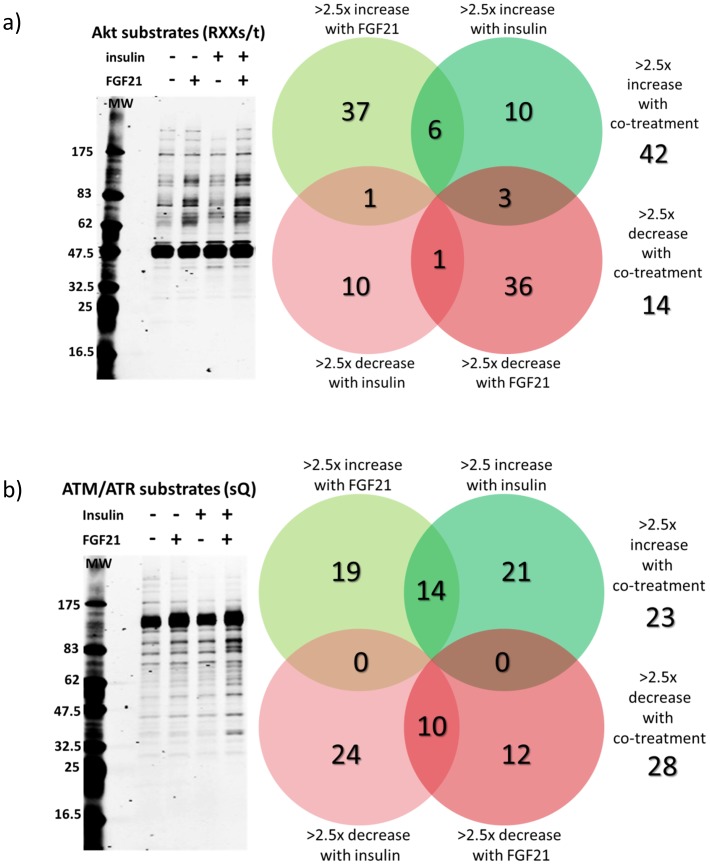
FGF21 and insulin synergistically induced phosphorylation of Akt substrates and ATM/ATR substrates. hASC-adipocytes were treated with FGF21 (10 nM) either alone or in combination with insulin (10 nM) for 30 min prior to harvest. hASC-adipocytes. **a**) Levels of phosphorylation of Akt substrates were assessed by western blot using a phospho-specific antibody recognizing proteins with the motif RXX(s/t). **b**) Levels of phosphorylation of ATM/ATR substrates were determined using a phospho-specific antibody recognizing proteins with the motif sQ.

To elucidate the signaling pathways that are involved in increasing synergistic glucose uptake by insulin and FGF21, we treated hASC-adipocytes in the absence and presence of FGF21 and with or without insulin for 30 min and used Cell Signaling Technology’s (CST’s) KinomeView service to screen for motif-specific antibodies that detected the largest changes in substrate phosphorylation in the co-treated samples, compared to control. Western blot analyses were performed to assess phosphorylation of S473-Akt and pERK1/2 as controls for insulin and FGF21 action respectively (Fig. S1a). Densitometric analyses of the western blots are shown in Fig. S1b. Additive effects were not observed for pAkt or pERK at this time point (30 min) Fig. S1a, but western blot analysis using the anti-phospho RXX(s/t) antibody and the anti-phospho sQ antibody revealed a synergistic increase in total phosphorylation of Akt substrates (Fig. 6a) and ATM/ATR substrates (Fig. 6b).

To identify the specific phosphoproteins that were synergistically induced by FGF21 and insulin, protein lysates from cells treated with FGF21 and insulin as described above were immunoprecipitated with the phospho-RXX(s/t) and phospho-sQ motif-specific antibodies, then analyzed by LC-MS/MS using CST’s PTMScan service. From a total 1465 non-redundant phosphorylated peptides identified with the phospho-RXX(s/t) antibody, FGF21 and insulin co-treatment yielded 42 proteins that had an increase in phosphorylation of more than 2.5-fold over basal while 14 proteins had a decrease in phosphorylation of more than 2.5-fold over basal (Fig. 6a). From 482 non-redundant phosphorylated peptides identified with the phospho-sQ antibody, FGF21 and insulin co-treatment yielded 23 proteins had an increase in phosphorylation of more than 2.5-fold over basal while 28 proteins had a decrease in phosphorylation of more than 2.5-fold over basal (Fig. 6b).

Out of the 42 Akt substrates with >2.5-fold increase in phosphorylation upon FGF21 and insulin co-treatment, 14 proteins were synergistically phosphorylated. The most synergistically phosphorylated proteins by FGF21/insulin co-treatment among the Akt substrates were pS264/T274-RSPRY1, pT353-PSRC2, pS194-DDI2, pT10/S12-lamin A/C, and pT328-FMIP (Table 1). Among the 14 Akt substrates with >2.5-fold decrease in phosphorylation upon FGF21 and insulin co-treatment, 3 proteins were synergistically dephosphorylated: pS250/S257-ARaf, pS257-RCOR1, and pY456/S466-DNMBP (Table 1).

**Table 1:** Akt substrates with synergistic increase and decrease in phosphorylation by FGF21/insulin co-treatment.

Gene	Protein	Phosphorylation site	Description	Accession	Peptide	FGF21	Ins	Combo
RSPRY1	RSPRY1	264, 274	RING finger and SPRY domain-containing protein 1 precursor	Q96DX4	LTIS*ESSISDRLVT*LESWANDPDYLKR	1.1	1.0	4.9
ZFC3H1	PSRC2	353	zinc finger C3H1 domain-containing protein	O60293	RIST*SDILSEK	2.2	−1.0	4.0
DDI2	DDI2	§194	protein DDI1 homolog 2	Q5TDH0	IRLFS*ADPFDLEAQAK	8.6	1.6	12.8
LMNA	lamin A/C	§10	prelamin-A/C	P02545	RAT*RSGAQASSTPLSPTR	−1.2	1.3	2.5
LMNA	lamin A/C	§12	prelamin-A/C	P02545	RATRS*GAQASSTPLSPTR	−1.2	1.3	2.5
THOC5	FMIP	§328	THO complex subunit 5 homolog	Q13769	RRRPT*LGVQLDDK	2.7	−1.1	3.6
UBR4	UBR4	§1762	E3 ubiquitin-protein ligase UBR4	Q5T4S7	ISESLVRHASTS*SPADK	2.1	−1.3	2.5
UBR4	UBR4	§1761	E3 ubiquitin-protein ligase UBR4	Q5T4S7	ISESLVRHAST*SSPADK	2.1	−1.3	2.5
AP1AR	AP1AR	174	AP-1 complex-associated regulatory protein	Q63HQ0	SSRLS*SDATVLTPNTESSCDLM#TK	7.6	1.1	10.3
AP1AR	AP1AR	§175	AP-1 complex-associated regulatory protein	Q63HQ0	SSRLSS*DATVLTPNTESSCDLM#TK	7.6	1.1	10.3
CALD1	caldesmon	546	caldesmon	Q05682	RRGET*ESEEFEK	−1.1	2.2	2.5
PHF3	PHF3	§1925	PHD finger protein 3	Q92576	FYS*DSHHLK	3.1	1.0	5.4
FRS2	FRS2	§503	fibroblast growth factor receptor substrate 2	Q8WU20	TRHNS*TDLPM	1.6	1.1	3.5
PDE3B	PDE3B	§295, §299	cGMP-inhibited 3,5-cyclic phosphodiesterase B	Q13370	RRS*SCVS*LGETAASYYGSCK	1.1	2.5	4.2
RPS6	S6	§235, §236, §241, §242	40S ribosomal protein S6	P62753	RRRLS*S*LRAST*S*K	4.0	4.0	8.4
SGTA	SGTA	§303	small glutamine-rich tetratricopeptide repeat-containing protein alpha	O43765	T*PSASNDDQQE	5.9	2.8	9.1
SGTA	SGTA	§303, §305	small glutamine-rich tetratricopeptide repeat-containing protein alpha	O43765	SQIRSRT*PS*ASNDDQQE	2.7	1.2	4.1
LRRC41	LRRC41	§327	leucine-rich repeat-containing protein 41	Q15345	RST*QESLTAGGTDLK	6.2	1.0	7.3
ARAF	A-Raf	§250, §257	serine/threonine-protein kinase A-Raf	P10398	GGS*DGTPRGS*PSPASVSSGRK	−2.9	−2.0	−8.7
RCOR1	RCOR1	§257	REST corepressor 1	Q9UKL0	EREES*EDELEEANGNNPIDIEVDQNK	−2.6	−1.6	−7.7
DNMBP	DNMBP	456, 466	dynamin-binding protein	Q6XZF7	RDY*ASLPPKRM#YS*QLK	−1.1	−1.4	−5.8

Legend: * - phosphorylation, # - oxidized methionine, § - published site.

Among the ATM/ATR substrates, only S210-Gab2 had significant synergy in protein phosphorylation by FGF21/insulin co-treatment (Table 2). Out of the 28 ATM/ATR substrates with >2.5-fold decrease in phosphorylation upon FGF21 and insulin co-treatment, 8 proteins were synergistically dephosphorylated, among which pS66/S68-AHCYL1, pT891-p300, pS513-FAM120A, and pS702.eIF4G were the most significant (Table 2). Confirmation of the LC-MS/MS phosphoproteomics results need to be conducted using phosphorylation site-specific antibodies, although these antibodies are not commercially available yet.

**Table 2:** ATM/ATR substrates with synergistic increase and decrease in phosphorylation by FGF21/insulin co-treatment.

Gene Name	Protein Name	Phosphorylation Site	Description	Accession	Peptide	FGF21	Insulin	Combo
GAB2	Gab2	§210	GRB2-associated-binding protein2 isoform b	Q9UQC2	SAS*FSQGTR	1.8	1.2	2.5
AHCYL1	AHCYL1	§68	putative adenosyl homocysteinase 2 isoform a	O43865	SIS*QSSTDSYSSAA	1.0	−1.5	−5.3
AHCYL1	AHCYL1	66	putative adenosyl homocysteinase 2 isoform a	O43865	S*ISQSSTDSYSSAA	1.0	−1.5	−5.3
EP300	p300	891	histone acetyltransferase p300	Q09472	QTPTPPTT*QLPQQVQPSLPAAPSADQPQQQPR	−1.2	−1.5	−5.4
MBLAC1	MBLAC1	242, §252	metallo-beta-lactamase domain-containing protein 1	A4D2B0	EAS*QPETEGGGNS*QQEPVVGDEEPALH	−1.6	−2.8	−6.7
FAM120A	FAM120A	513	constitutive coactivator of PPAR-gamma-like protein 1	Q9NZB2	AEGSSTASSGS*QLAEGK	−1.2	−1.6	−2.9
EIF4G1	eIF4G	§704	eukaryotic translation initiation factor 4 gamma 1 isoform 6	Q04637	GPAGLGPRRS*QQGPR	1.2	−1.7	−2.7
EXOC2	Sec5	422	exocyst complex component 2	Q96KP1	GNPGLHSPM#LDLDNDTRPSVLGHLS*QTASLK	−2.7	−1.6	−4.1
EXOC2	Sec5	424	exocyst complex component 2	Q96KP1	GNPGLHSPM#LDLDNDTRPSVLGHLSQT*ASLK	−2.7	−1.6	−4.1
RASAL2	RASAL2	734	ras GTPase-activating protein nGAP isoform 1	Q9UJF2	LDVPIRLT*GSQLSITQVASIK	−1.6	−1.3	−2.6
FASN	FASN	2465	fatty acid synthase	P49327	TGGAYGEDLGADYNLS*QVCDGK	−1.8	−1.9	−2.8

Legend: * - phosphorylation, # - oxidized methionine, § - published site hASC-adipocytes were treated with FGF21 (10 nM) either alone or in combination with insulin (10 nM) for 30 min prior to harvest. a) Lysates were subject to western blot for pAkt and pERK. b) Densitometric quantification of western blots.

## Discussion

FGF21 has emerged as a promising therapeutic molecule for the treatment of metabolic diseases [7], [16]. Adipose tissue is one of the primary direct target tissues for FGF21 action since both the receptor FGFR1 and co-receptor KLB are expressed in these cells [14]. Although rodent studies have demonstrated FGF21-mediated glucose uptake in adipocytes and in adipose tissue, there are no reports that interrogate acute FGF21 signaling in human adipocyte cultures. In this study, we used a previously established in vitro cell culture system that resembles human adipocytes [18]. We performed gene expression analyses in these cells and from subcutaneous and visceral adipose tissue from lean healthy, obese non diabetic, and obese subjects with T2D. We confirmed that both hASC-adipocytes and human adipose tissue express both FGFR1 and KLB and that the ratio of KLB/FGFR1 is similar between human adipose tissue and hASC-adipocytes, suggesting the hASC-adipocytes are a good in vitro system that resembles human fat cells and enabling proper translation potential from these cells to human adipose tissue.

In vivo studies have shown robust acute glucose lowering ability of FGF21, the effects of which are driven in part by glucose uptake in adipose tissue [14]. We confirmed that in hASC-adipocytes FGF21 causes increase of glucose uptake and that this effect is significantly attenuated in presence of a neutralizing antibody, suggesting specificity of the effect. Moreover, FGF21 alone does not increase phosphorylation of InsR, IGF1R, IRS1, but increases Akt phosphorylation in the absence of insulin. Importantly, Akt phosphorylation is increased by FGF21 and insulin additively, suggesting that the mechanism for this effect lies at, or below Akt.

An interesting finding of our study is that FGF21 synergistically increases phosphorylation of STAT3 in adipose tissue in presence of leptin. Adipocyte STAT3 contributes to regulation of body weight homeostasis in part through direct effects of leptin on adipocytes [21]. Weight loss in obese animal models upon FGF21 administration is driven, in part by leptin since weight loss upon FGF21 treatment in ob/ob and db/db mice is completely abolished [22], [23]. Moreover, FGF21 administration in DIO mice decreases circulating leptin and increases LepRb expression in liver and epididymal white adipose tissue [24]. Since leptin is a key component of FGF21 action, our data opens up the intriguing possibility that FGF21 can independently activate STAT3 in the adipose tissue, making these cells more leptin-responsive. Further studies are required to understand the crosstalk between FGF21 and leptin pathway.

FGF21 treatment in hASC-adipocytes also caused a modest, but significant increase in AMPK phosphorylation, which is consistent with reports suggesting activated AMPK is required for enhanced oxidative metabolism [25]. Recent evidence indicates an important role for AMPK in brown fat [26] and white fat function [25], [27]. However, the role of AMPK in promoting energy utilization in adipose tissue remains unclear. A mechanistic understanding of this process could help uncover novel pathways to promote burning of fat.

Although FGF21 drives its metabolic effects through interaction with FGFR1c and KLB [8], [9], the precise sequence of interactions is unclear. However, in vitro studies have shown that the C-terminal region of FGF21 interacts with KLB to trigger signaling events [28]. All these studies have relied on in vitro assays to measure in vitro potency. To confirm that the FGF21 effects were being mediated with its interaction with KLB, we used a C-terminal truncated FGF21 that lacks the last 10 amino acids (FGF21-dC10) and demonstrated that there is a 50-fold shift in EC_50_ values compared to native FGF21. This data is consistent with previous studies that have reported a right-shift in EC_50_ values upon C-terminal truncation [19].

An important observation in this study is that FGF21 increases basal glucose uptake in hASC-adipocytes, an effect that is KLB dependent. These results are consistent with results from FGF21 in 3T3L1 cells [1], [14] and opens up the possibility of FGF21 as a glucose lowering therapeutic in new onset type 1 diabetes. Indeed, administration of FGF21 in STZ-treated mice lowers blood glucose [29], likely in part by increasing uptake of glucose in fat cells. Data from our hASC-adipocytes shows the translatability of these findings in man. Further studies are required to tease out this important observation to establish FGF21 pharmacology in a type 1 patient population.

Importantly, we show that FGF21 synergizes with insulin to improve insulin signaling in hASC-adipocytes resulting in a functional response of glucose uptake in these cells. Further, in presence of FGF21 the amount of insulin required to elicit similar glucose uptake is decreased by 10-fold, suggesting insulin sensitization in the adipocytes upon FGF21 administration. This observation is consistent with the clinical study with FGF21 where fasting insulin was decreased approximately 40% at the highest dose, with about 5% glucose lowering from baseline [17]. While in a physiological setting the actions of FGF21 are more complex, data from hASC-adipocytes captures a snapshot of physiological processes.

In a previous study, more than 130 phosphoproteins involved in metabolic pathways such as glucose uptake, insulin receptor signaling, Erk/Mapk signaling cascades and lipid metabolism were identified that were modulated greater than 1.5 fold by FGF21 treatment in 3T3-L1 adipocytes [30]. As expected, this study identified several novel proteins that are yet to be linked to FGF21 pharmacology [30]. Since Akt phosphorylation was synergistically increased by FGF21 and insulin in hASC-adipocytes, we performed in-depth phosphoproteomic analyses using phospho-RXX(s/t) and phospho-sQ motif-specific antibodies, following analyses by LC-MS/MS, and identified several novel proteins that had changes in phosphorylation levels in the absence and presence of insulin or FGF21. For stratification, we report changes that are greater than 2.5-fold compared to control. We identified several new proteins linked to FGF21 pharmacology in the adipose tissue. Further studies are required to establish and determine the bona-fide physiological contribution of one or more of these proteins to the beneficial metabolic effects of FGF21.

In conclusion, FGF21 has emerged as an exciting new therapeutic agent that exhibits robust efficacy in preclinical species and man. Despite a large body of work, the mechanisms underlying the metabolic effects of pharmacological FGF21 administration remains to be precisely elucidated. However, adipose tissue is a key target mediating FGF21 action, along with the CNS [31]. Further studies are required to elucidate and tease apart the contribution of these two tissues leading to weight loss and other metabolic benefits. In the next few years, results from clinical trials with the next generation of FGF21 analogs with extended half-lives may help unravel the complexities of this pathway.

## Methods

### Recombinant Proteins

Recombinant human beta-klotho (KLB) was purchased from R&D Systems (5889-KB-050). Insulin was purchased from Sigma (I0516). Wild type FGF21 and FGF21-dC10 were generated in house.

### Human Adipocyte Culture

Human subcutaneous adipose stem cells (hASC) were purchased from Zenbio (lot# 062801) and grown up to 6 passages in growth media containing DMEM/F12 with 10% v/v FBS, 5 ng/ml hEGF (Invitrogen 13247-051), 1 ng/ml bFGF (Invitrogen 13256-029), and 0.25 ng/ml hTGF (Sigma T1654). For differentiation, cells were plated at 7500 cells/cm^2^ and maintained in growth media for 3 days, then changed to preadipocyte media (Promocell C27410) and maintained for another 4 days. Media was then changed to differentiation media (Zenbio DM-2), maintained for 7 days, then changed to a 1∶1 mix of DM-2 and adipocyte maintenance media (Zenbio AM-1) for another 7 days. Prior to any assay, cells were serum and insulin starved for 24 hours in 0.2% BSA (Sigma A7888) w/v in DMEM/F12 (Life Technologies). All culture incubations were done at 37°C with 5% CO_2_.

### Human adipose tissue collection

In accordance with the Declaration of Helsinki, human adipose tissue was collected from subjects in a study that was reviewed and approved by the Tufts University Health Sciences IRB (IRB # 10211). Subjects provided written consent for the use of their tissue for research that would otherwise have been discarded following surgery.

### hASCs were induced to differentiate into lipid-containing adipocytes

2,400 cells per well of hASCs cultured in 96-well culture plates for 3 days in custom growth media and 4 days in PromoCell preadipocyte media, then imaged at 10× magnification: A) immediately prior to differentiation, or B) after 14 total days of adipogenic differentiation, with 7 days in DM-2 and 7 days in AM-1:DM-2, and 1 day of serum/insulin deprivation, in which the cells were used for various functional assays.

### Gene Expression and copy number

RNA was extracted from cells using the Qiagen RNeasy 96 kit (Qiagen 74181) including a DNaseI digestion step (Qiagen 79254). Reverse transcription was done using TaqMan Reverse Transcription Reagents (Life Technologies N8080234). Quantitative real-time PCR was done with Taqman Universal PCR Master Mix (Life Technologies 4304437) using gene specific probes from Life Technologies for: hKLB (Hs00545621_m1), hTBP (Hs00427620_m1), hFGFR1c (custom made based on sequences reported.

For copy number, serial dilution of FGFR1 or KLB plasmid DNA was used to generate a standard curve for qPCR, and copy number was calculated based on the molecular weight of the plasmid. The copy numbers of the standards used to generate a curve for FGFR1 are as follows, 3.8E-08, 1.9E-08, 9.5E-09, 4.8E-09, 2.4E-09, 1.2E-09, 6.0E-10, 3.0E-10, 1.5E-10, 7.5E-11, 3.7E-11. The copy numbers of the standards used to generate a curve for KLB are as follows, 3.0E-07, 1.5E-07, 7.4E-08, 3.7E-08, 1.9E-08, 9.0E-09, 4.6E-09, 2.3E-09, 1.2E-09, 5.8E-10.

2.9E-10. Copy number was determined using the following equation: Calculated DNA by linearized plasmid/[(MW of Nucleic acid X plasmid bps)/6.02×10^23^].

### In vitro Glucose Uptake Assay

In vitro glucose uptake in hASC-adipocytes was modified from [32]. hASC-adipocytes were stimulated with insulin and/or FGF21 in 0.2%BSA w/v in DPBS (Life Technologies 14040133) for 30 min at 37°C. After stimulation, media was supplemented with 28 uM [^14^C]-2DG (PerkinElmer, NEC-495A), 800 uM 2DG (Sigma Cat# D3179) at final concentration for 30 min at 37°C. Cells were washed three times with cold PBS then lysed with 100 uL 1% Triton X-100 w/v in ddH_2_O for 30 min at room temp while shaking. Lysates were transferred to a 96-well LumaPlate (Perkin Elmer 6005630), dried overnight, and read for 14C on a Perkin Elmer Trilux MicroBeta for CPM. FGF21 neutralizing antibody (nAb) was custom generated and purified (clone# 9A2.H8.G4) by Green Mountain Antibodies using peptide, CGGPSQGRSPSYAS, for immunization. 100 ug/ml neutralizing antibody was used to neutralize 32 nM FGF21 in assay buffer 30 min prior to treating the cells.

### Phosphoproteomic and PathScan assays

For Meso Scale Discoveries (MSD) assays, adipocytes were stimulated with insulin and/or FGF21 for 5 min to 10 min at 37°C, as indicated in the figures, and lysed for 30 min on ice with respective lysis buffer included in the assay kits: pIR, pIRS1, pIGF1R (MSD K11151C); tIR, tIRS1, tIGF1R (MSD K11152C); pS473-Akt/tAkt (MSD K15100D); pERK/tERK (MSD K151DWD). MSD assays were performed as recommended by the manufacturer. EC50 curves were generated using GraphPad Prism 5.0, and p-values were calculated by student’s t-test. For Cell Signaling Technologies (CST) KinomeView and PTMScan, adipocytes were stimulated with insulin and/or FGF21 for 30 min at 37°C, and lysed for 30 min on ice. For KinomeView lysates were prepared and processed for western blotting with various motif-specific CST antibodies as previously described [33]. 10 µg protein was loaded per well. For CST PTMScan, lysates were prepared and processed for LC-MS/MS analysis as previously described [33]. 10 mg total protein was immunoaffinity-purified with anti-phospho-RXX(s/t) antibody for Akt substrates (CST 9614) or anti-phospho-(s)Q antibody for ATM/ATR substrates. Control western blots were done using primary antibodies for pERK/pAkt (CST 5301) and β-actin (CST 4967).

The PathScan Intracellular Signaling Array Kit (CST#7323) was purchased from Cell Signaling Technology. Target capture antibodies have been spotted on these plates that allow simultaneous detection of 18 important and well-characterized signaling molecules when phosphorylated or cleaved. hASC-adipocytes were lysed and processed according to manufacturer’s protocol. Representative images were captured (not shown) using a digital imaging system. The spot intensity of captured images was quantified using standard array analysis software.

## Supporting Information

Figure S1 hASC-adipocytes were treated with FGF21 (10 nM) either alone or in combination with insulin (10 nM) for 30 min prior to harvest. a) Lysates were subject to western blot for pAkt and pERK. b) Densitometric quantification of western blots.
(TIF)

## References

[1] Kharitonenkov A, Shiyanova TL, Koester A, Ford AM, Micanovic R, et al (2005) FGF-21 as a novel metabolic regulator. J Clin Invest 115: 1627–1635. 15902306 10.1172/JCI23606 PMC1088017

[2] Fon Tacer K, Bookout AL, Ding X, Kurosu H, John GB, et al (2010) Research resource: Comprehensive expression atlas of the fibroblast growth factor system in adult mouse. Mol Endocrinol 24: 2050–2064. 20667984 10.1210/me.2010-0142 PMC2954642

[3] Kliewer SA, Mangelsdorf DJ (2010) Fibroblast growth factor 21: from pharmacology to physiology. Am J Clin Nutr 91: 254S–257S. 19906798 10.3945/ajcn.2009.28449B PMC2793111

[4] Xu J, Lloyd DJ, Hale C, Stanislaus S, Chen M, et al (2009) Fibroblast growth factor 21 reverses hepatic steatosis, increases energy expenditure, and improves insulin sensitivity in diet-induced obese mice. Diabetes 58: 250–259. 18840786 10.2337/db08-0392 PMC2606881

[5] Kharitonenkov A, Beals JM, Micanovic R, Strifler BA, Rathnachalam R, et al (2013) Rational design of a fibroblast growth factor 21-based clinical candidate, LY2405319. PLoS One 8: e58575. 23536797 10.1371/journal.pone.0058575 PMC3594191

[6] Coskun T, Bina HA, Schneider MA, Dunbar JD, Hu CC, et al (2008) Fibroblast growth factor 21 corrects obesity in mice. Endocrinology 149: 6018–6027. 18687777 10.1210/en.2008-0816

[7] Huang J, Ishino T, Chen G, Rolzin P, Osothprarop TF, et al (2013) Development of a Novel Long-Acting Antidiabetic FGF21 Mimetic by Targeted Conjugation to a Scaffold Antibody. J Pharmacol Exp Ther 346: 270–280. 23720456 10.1124/jpet.113.204420

[8] Ding X, Boney-Montoya J, Owen BM, Bookout AL, Coate KC, et al (2012) betaKlotho is required for fibroblast growth factor 21 effects on growth and metabolism. Cell Metab 16: 387–393. 22958921 10.1016/j.cmet.2012.08.002 PMC3447537

[9] Foltz IN, Hu S, King C, Wu X, Yang C, et al (2012) Treating diabetes and obesity with an FGF21-mimetic antibody activating the betaKlotho/FGFR1c receptor complex. Sci Transl Med 4: 162ra153. 10.1126/scitranslmed.3004690 23197570

[10] Veniant MM, Hale C, Helmering J, Chen MM, Stanislaus S, et al (2012) FGF21 promotes metabolic homeostasis via white adipose and leptin in mice. PLoS One 7: e40164. 22792234 10.1371/journal.pone.0040164 PMC3391219

[11] Yang C, Jin C, Li X, Wang F, McKeehan WL, et al (2012) Differential specificity of endocrine FGF19 and FGF21 to FGFR1 and FGFR4 in complex with KLB. PLoS One 7: e33870. 22442730 10.1371/journal.pone.0033870 PMC3307775

[12] Holland WL, Adams AC, Brozinick JT, Bui HH, Miyauchi Y, et al (2013) An FGF21-adiponectin-ceramide axis controls energy expenditure and insulin action in mice. Cell Metab 17: 790–797. 23663742 10.1016/j.cmet.2013.03.019 PMC3667496

[13] Lin Z, Tian H, Lam KS, Lin S, Hoo RC, et al (2013) Adiponectin mediates the metabolic effects of FGF21 on glucose homeostasis and insulin sensitivity in mice. Cell Metab 17: 779–789. 23663741 10.1016/j.cmet.2013.04.005

[14] Xu J, Stanislaus S, Chinookoswong N, Lau YY, Hager T, et al (2009) Acute glucose-lowering and insulin-sensitizing action of FGF21 in insulin-resistant mouse models-association with liver and adipose tissue effects. Am J Physiol Endocrinol Metab 297: E1105–1114. 19706786 10.1152/ajpendo.00348.2009

[15] Arner P, Pettersson A, Mitchell PJ, Dunbar JD, Kharitonenkov A, et al (2008) FGF21 attenuates lipolysis in human adipocytes - a possible link to improved insulin sensitivity. FEBS Lett 582: 1725–1730. 18460341 10.1016/j.febslet.2008.04.038

[16] Reitman ML (2013) FGF21 mimetic shows therapeutic promise. Cell Metab 18: 307–309. 24011067 10.1016/j.cmet.2013.08.014 PMC3789140

[17] Gaich G, Chien JY, Fu H, Glass LC, Deeg MA, et al (2013) The effects of LY2405319, an FGF21 analog, in obese human subjects with type 2 diabetes. Cell Metab 18: 333–340. 24011069 10.1016/j.cmet.2013.08.005

[18] Bordicchia M, Liu D, Amri EZ, Ailhaud G, Dessi-Fulgheri P, et al (2012) Cardiac natriuretic peptides act via p38 MAPK to induce the brown fat thermogenic program in mouse and human adipocytes. J Clin Invest 122: 1022–1036. 22307324 10.1172/JCI59701 PMC3287224

[19] Micanovic R, Raches DW, Dunbar JD, Driver DA, Bina HA, et al (2009) Different roles of N- and C- termini in the functional activity of FGF21. J Cell Physiol 219: 227–234. 19117008 10.1002/jcp.21675

[20] Yie J, Hecht R, Patel J, Stevens J, Wang W, et al (2009) FGF21 N- and C-termini play different roles in receptor interaction and activation. FEBS Lett 583: 19–24. 19059246 10.1016/j.febslet.2008.11.023

[21] Cernkovich ER, Deng J, Bond MC, Combs TP, Harp JB (2008) Adipose-specific disruption of signal transducer and activator of transcription 3 increases body weight and adiposity. Endocrinology 149: 1581–1590. 18096662 10.1210/en.2007-1148 PMC2276706

[22] Kim HW, Lee JE, Cha JJ, Hyun YY, Kim JE, et al (2013) Fibroblast growth factor 21 improves insulin resistance and ameliorates renal injury in db/db mice. Endocrinology 154: 3366–3376. 23825123 10.1210/en.2012-2276

[23] Adams AC, Coskun T, Rovira AR, Schneider MA, Raches DW, et al (2012) Fundamentals of FGF19 & FGF21 action in vitro and in vivo. PLoS One 7: e38438. 22675463 10.1371/journal.pone.0038438 PMC3365001

[24] Adams AC, Cheng CC, Coskun T, Kharitonenkov A (2012) FGF21 requires betaklotho to act in vivo. PLoS One 7: e49977. 23209629 10.1371/journal.pone.0049977 PMC3507945

[25] Chau MD, Gao J, Yang Q, Wu Z, Gromada J (2010) Fibroblast growth factor 21 regulates energy metabolism by activating the AMPK-SIRT1-PGC-1alpha pathway. Proc Natl Acad Sci U S A 107: 12553–12558. 20616029 10.1073/pnas.1006962107 PMC2906565

[26] Mulligan JD, Gonzalez AA, Stewart AM, Carey HV, Saupe KW (2007) Upregulation of AMPK during cold exposure occurs via distinct mechanisms in brown and white adipose tissue of the mouse. J Physiol 580: 677–684. 17272339 10.1113/jphysiol.2007.128652 PMC2075554

[27] Koh HJ, Hirshman MF, He H, Li Y, Manabe Y, et al (2007) Adrenaline is a critical mediator of acute exercise-induced AMP-activated protein kinase activation in adipocytes. Biochem J 403: 473–481. 17253964 10.1042/BJ20061479 PMC1876380

[28] Yie J, Wang W, Deng L, Tam LT, Stevens J, et al (2012) Understanding the physical interactions in the FGF21/FGFR/beta-Klotho complex: structural requirements and implications in FGF21 signaling. Chem Biol Drug Des 79: 398–410. 22248288 10.1111/j.1747-0285.2012.01325.x

[29] STANISLAUS S, Xu J, Ellison MM (2013) Method of treating or ameliorating type 1 diabetes using fgf21. Google Patents.

[30] Muise ES, Souza S, Chi A, Tan Y, Zhao X, et al (2013) Downstream signaling pathways in mouse adipose tissues following acute in vivo administration of fibroblast growth factor 21. PLoS One 8: e73011. 24039848 10.1371/journal.pone.0073011 PMC3765203

[31] Owen BM, Ding X, Morgan DA, Coate KC, Bookout AL, et al.. (2014) FGF21 Acts Centrally to Induce Sympathetic Nerve Activity, Energy Expenditure, and Weight Loss. Cell Metab. 10.1016/j.cmet.2014.07.012 PMC4192037 25130400

[32] Talukdar S, Oh da Y, Bandyopadhyay G, Li D, Xu J, et al (2012) Neutrophils mediate insulin resistance in mice fed a high-fat diet through secreted elastase. Nat Med 18: 1407–1412. 22863787 10.1038/nm.2885 PMC3491143

[33] Stokes MP, Farnsworth CL, Moritz A, Silva JC, Jia X, et al (2012) PTMScan direct: identification and quantification of peptides from critical signaling proteins by immunoaffinity enrichment coupled with LC-MS/MS. Mol Cell Proteomics 11: 187–201. 22322096 10.1074/mcp.M111.015883 PMC3418847

