# Supplementary figures and images for: Fibroblast Growth Factor 21 Improves Insulin Sensitivity and Synergizes with Insulin in Human Adipose Stem Cell-Derived (hASC) Adipocytes
Source: PLoS One. 2014 Nov 3;9(11):e111767. doi: 10.1371/journal.pone.0111767 (PMC4218812; doi:10.1371/journal.pone.0111767)

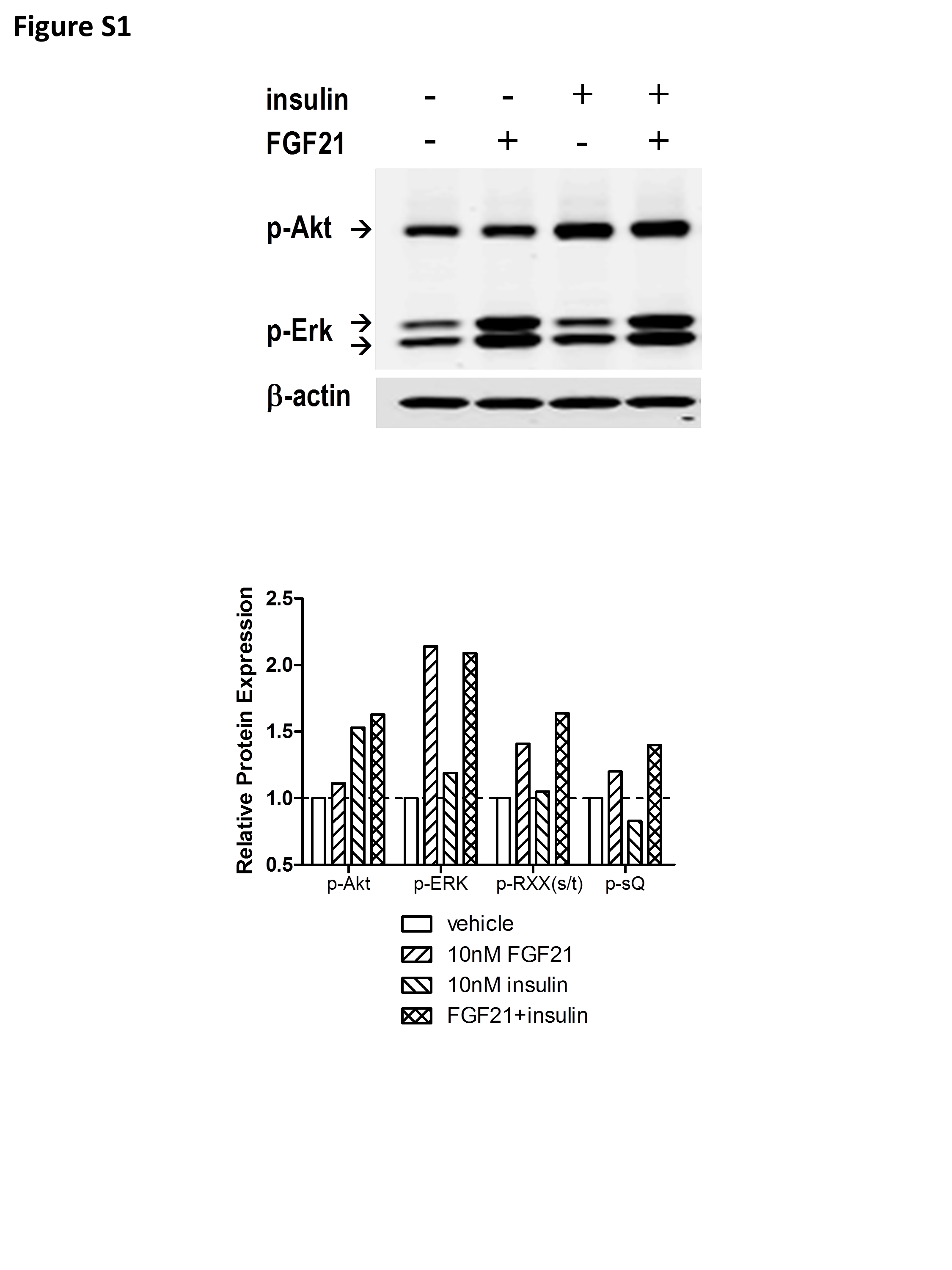

Supplement: Figure S1 — hASC-adipocytes were treated with FGF21 (10 nM) either alone or in combination with insulin (10 nM) for 30 min prior to harvest. a) Lysates were subject to western blot for pAkt and pERK. b) Densitometric quantification of western blots. (TIF) [file pone.0111767.s001.tif]
